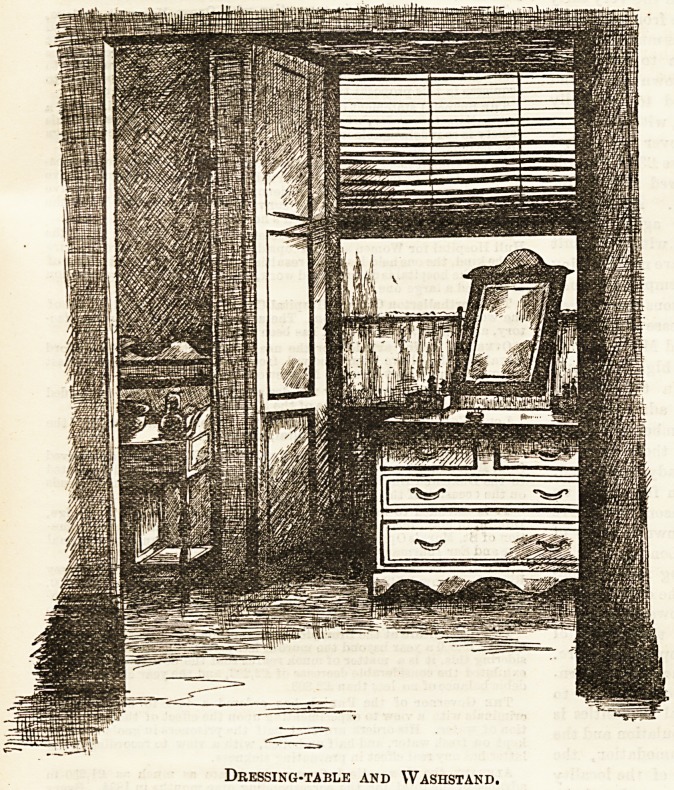# Practical Departments

**Published:** 1895-12-14

**Authors:** 


### PRACTICAL DEPARTMENTS.

### III.—FURNITURE AND FITTINGS FOR NURSES' HOMES.

One of the latest developments in nurse training, in the London nursing world at least, is the establishment of a " Preliminary Training Home " for probationers in connection with the London Hospital. Tredegar House, a substantial, somewhat old-fashioned building, fronting on Bow Road, a mile or so east of the hospital, has lent itself well to adaptation to its present purpose, and the rooms have been excellently fitted up, the furniture being much the same as in the Hospital Nursing Home, with certain improvements. The washstand, for instance, is a better pattern—a portion of it may be seen in the accompanying sketch—with marble slab and convenient little cupboard. The " combination " dressing-table and chest of drawers is a useful size, and, as well as the rest of the furniture, all of which has been supplied by Messrs. Debenham and Freebody, is good and well made. The choice of colouring throughout has been very happy, even to the smallest details. It costs no more—in money, at least—to see that harmony shall reign in such matters, only a considerable expenditure of time and trouble, but these have been well laid out in the present case, and the result is excellent, and most pleasant to the eye.

As a proof of what may be done to make the best of a building not intended for such a purpose, Tredegar House is a bright example. Built long before the days of universal bath-rooms, these necessaries of modern life have been ingeniously created on each floor by the conversion of a dressing-room, and the putting up of a partition to form a passage. There are also well-flushed slop-sinks of the most approved

Dressing-table and Washstand.

pattern, and fixed tip-up wash-hand basins on each floor, the latter arrangement obviating the too constant emptying of bedroom slops. The study and sitting-room and dining-room are simply and comfortably furnished; pantry and kitchen are amply stocked. Instruction in the practical details of sweeping and cleaning forming a considerable portion of the preliminary training scheme, there is plentiful provision made in this line; for classes and lectures a large room has been contrived in the garden from what was in past days a fern-house. Though some nursing authorities cling to the older fashion, and hold a scheme of this kind to be a beginning at the wrong end, in preparing Tredegar House for the reception of probationers it must be acknowledged by all that the London Hospital Committee have carried out the practical details most thoroughly and completely, and in their admirable arrangements for the due comfort of their nurses in their special quarters lead the way amongst London nursing homes.

In the case of two London hospitals at the present time more accommodation is being arranged for nurses. The authorities of St. George's Hospital are building, and not before it was wanted, a home, at some little distance from the hospital, for their at present very inadequately housed staff, and a new wing is about to be added to the present home at the London Hospital. Here, therefore, will be scope for carrying out the best and most practical of modern suggestions with regard to such buildings. It is unfortunate when the nursing staff of a hospital have any distance to go to reach their quarters; but in the case of St. George's Hospital, under existing circumstances, this could not well be avoided.

The West Ham Hospital has just been enlarged and improved, particularly in respect to the accommodation for the nursing staff, which is now all it ought to be. The topmost floor of the newly-erected wing is devoted to rooms for the day nurses. Very pleasant, airy little rooms they are, and comfortably furnished; and the sitting-room, with its piano and easy chairs, has an exceptionally cosy aspect. The night nurses' quarters are, as they should always be, entirely separate, and shut off by three doors from the hospital proper, so as to ensure a maximum of quiet during sleeping hours. In too many cases the utter importance of securing absolute silence in the neighbourhood of night nurses' rooms receives far too small a share of attention. Most people, in spite of custom, find it more difficult to sleep in the day-time than during the usual hours, and are much more easily disturbed by slight sounds. In new buildings for the accommodation of nurses it should be a matter of the first consideration to so place the rooms for the night staff that no slightest interference with their hard-earned rest shall be possible.

A word must be said with regard to the nursing homes of some of the latest-built workhouse infirmaries. These are many of them excellent in planning and construction, as well as in furnishing generally. Separate and very fair-sized rooms are provided for each nurse, quite simply fitted, but in those we have seen the furniture is very good of its kind; neat iron spring-mattressed beds with hair mattresses convenient wardrobe, and dressing-table, washstand, &c., leaving just room enough for their occupants to move freely. The floors are stained and polished, with the addition of a small piece of carpet or mat beside the bed. At the St. Olave's Union Infirmary the nursing home is exceedingly comfortable, and there are even special tennis courts for the nurses, a luxury by no means to be found at every hospital. The Lewisham Infirmary Nursing Home is another example, and there are others, notably those of the Marylebone and St. Pancras Infirmaries, which are in many respects models, structurally and practically, of what nursing homes ought to be.

## Figures and Tables

**Figure f1:**